# Computational Study of Coordinated Ni(II) Complex with High Nitrogen Content Ligands

**DOI:** 10.5402/2011/920753

**Published:** 2011-04-27

**Authors:** Bo Tang, Jia-Hai Ye, Xue-Hai Ju

**Affiliations:** School of Chemical Engineering, Nanjing University of Science and Technology, Nanjing 210094, China

## Abstract

Density functional computations were performed on two tetracoordinated Ni(II) complexes as high nitrogen content energetic materials (**1**: dinickel bishydrazine ter[(1*H*-Tetrazol-3-yl)methan-3yl]-1*H*-tetrazole and **2**: dinickel tetraazide ter[(1*H*-Tetrazol-3-yl)methan-3yl]-1*H*-tetrazolate). The geometrical structures, relative stabilities and sensitivities, and thermodynamic properties of the complexes were investigated. The energy gaps of frontier molecular orbital (HOMO and LUMO) and vibrational spectroscopies were also examined. There are minor Jahn-Teller distortions in both complexes **1** and **2**, with two long Ni–N bond lengths and two short ones. The enthalpies of combustion for both complexes are over 3600 kJ/mol. The N–N bond lengths in the moieties of hydrazine and azide ligands increase in the coordination process compared to those of the isolated molecules.

## 1. Introduction

The development of weapon ammunition and energetic materials requires higher integrate performance including powerful efficacy, controllable energy release, insensitivity, and being friendly to environment. However, conventional energetic materials cannot fulfill all of these requests simultaneously. High energy density materials (HEDM) offer the needful properties mentioned above fortunately, with additional advantages of safety and low characteristic signal [1–6]. A key property for the design and synthesis of new energetic materials is the heat of formation (HOF) [7], which is used to assess potential performance in technology applications. Since measuring HOF as well as other essential information through experimental sample is inaccessible and dangerous for HEDM, thus we resort to theoretical methods. Therefore, discovery and exploitation of new types of HEDM through theoretical evaluations are our focus in this paper.

In the latest decades, investigators started to consider transition metal as the component of energetic materials because transition metal possesses numerous excellences [8–12]. Herein, we chose transition metal nickel as the object and emphasized nitrogen-coordinated Ni(II) complex. Among multitudinous complexes containing transition metal, polynuclear transition metal complexes are rather noticeable, interesting both from a fundamental and from an application viewpoint. In previous literatures, N_3_^−^, phenoxo and di-2-pyridyl ketone have been used to bridge the center metal ion Ni(II) [11–15]. For example, all the Ni atoms in [Ni_6_(CO_3_)(N_3_)_6_{pyCOpyC(O)(OMe)py}_3_ (MeOH)_3_](ClO_4_)_2_ are hexacoordinate [14], exhibiting distorted octahedral coordination geometries. While in some other complexes, the Ni(II) exists as the form of tetracoordinate, by which Ni(II) coordinates with either four nitrogen atoms on a plane or with three nitrogen atoms and one sulfur exhibiting pseudotetrahedron [16, 17]. 

**Figure 1:**
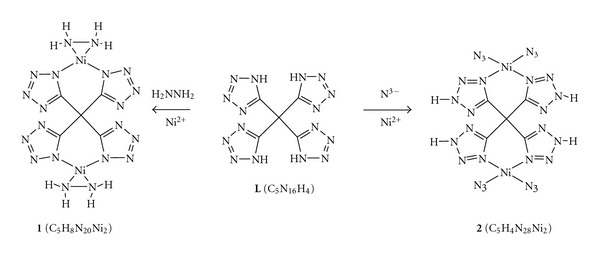
Molecular structures of **1**, **2,** and **L** (1): dinickel bishydrazine ter[(1*H*-Tetrazol-3-yl)methan-3yl]-1*H*-tetrazole, (2): dinickel tetraazide ter[(1*H*-Tetrazol-3-yl)methan-3yl]-1*H*-tetrazolate, and **(L):** tetra(1*H*-Tetrazol-5-yl)methane.

A variety of derivatives-involve tetrazole have recently been synthesized in experiment [18, 19] and verified a good selectivity for coordination with Ni(II). Consequently tetrazole with four potential coordinated nitrogen atoms which are ready to bridge transition metals of our required binuclear transition metal complexes, become the candidate group. In order to expand the magnitude of nitrogen content and diversification of bridging situation, we used tetrazole derivatives to accomplish the bridging of two Ni(II) into complexes. With four tetrazoles substituting the hydrogens on the methane in a manner that the C-substituted tetrazoles are more stable than the corresponding N-substituted isomers [7], the ligand **L** (Figure 1) was produced. Compared to the single tetrazole, each **L **has four times of nitrogen atoms to coordinate with Ni(II).

One thing that has to be paid attention to is that **L** can exist in three kinds of forms towards tetrazoles: tetrazoles, tetrazolates, and tetrazolium (namely neutral, deprotonated and protonated tetrazoles). Considering the interaction with positive metal ion of Ni(II), previous two forms are preferentially employed to coordinate with Ni(II). In the light of distinguishing form of **L**, we should have two other groups to coordinate with Ni(II) separately, that is, a neutral or a negatively charged group. To add more nitrogen content into the complexes, the N_2_H_4_ and N_3_^−^ were selected as additional ligands. Accordingly, the target compounds are **1 **and **2** as shown in Figure 1. We optimized the structures of **1 **and **2** with DFT-B3LYP method. The infrared spectroscopy was simulated. The changes of standard thermodynamic properties in the coordinating reactions were predicted, the stability constants were evaluated from the changes of standard Gibbs free energies. The combustion heats were calculated for the title complexes. Finally, the energy gaps were used to evaluate the sensitivity of the complexes. These results are beneficial for experimental investigators to advance thorough researches on these metallic complexes with high nitrogen content. 

## 2. Computational Methods

The density functional B3LYP can produce accurately and economically the heats of formation for compounds containing tetrazole or transition metal [7, 20]. Previous researches also indicated that B3LYP is one of the best choices for metallic complexes [21, 22]. Many kinds of basis sets were taken into consideration for comparison. The CEP-31G, LanL2DZ, and SDD pseudopotential basis sets are commonly used for metallic atom and 6-31+G** and 3-21+G* for nonmetallic atoms. The Ni(N_3_)_4_^2−^ was used as a benchmark to see if the outcome is consistent with experimental data. At the beginning, we tried all the combinations of the above-mentioned basis sets. As a result, only the combination of SDD with 6-31+G** or 3-21+G* produced the convergence structures. We obtained the following optimized bond lengths and angles of Ni(N_3_)_4_^2−^ when the combination of SDD with 3-21+G* was used: *θ*(N–N–N) = 166.71°, 167.61°, 171.11° and 177.81°. r(Ni–N) = 2.0341 Å, 2.0431 Å, 2.0281 Å, and 1.9731 Å. Both the bond lengths and angles are in good agreement to the experimental values of the azide-coordinated Ni(II) complex [Ni_2_L_2_(N_3_)_2_(H_2_O)_2_], whose *θ*(N–N–N) = 178.4(5)°, and r(Ni–N) are in range of 2.028–2.096 Å [12]. In addition, calculated bond lengths are also in good agreement and the experimental *r*(Ni–N) = 1.995 Å and 2.001 Å in the tetracoordinate Ni(II) complex [17]. The good agreement between our calculated structure of isolated Ni(N_3_)_4_^2−^ molecule with the experiment made us confident to proceed to the next step: optimization for title compound with DFT-B3LYP method. 

Ligand **L**, hydrazine, azide anion, and the coordinated Ni complexes generated from the Chem3D were fully optimized at the DFT-B3LYP level by the Berny method [23] with basis sets of SDD for Ni atom and 3-21+G* for nonmetallic atoms. The computations were performed with the Gaussian 03 package [24] at the B3LYP level. The optimizations were performed without any symmetry restrictions using the default convergence criteria in the programs. All of the optimized structures were characterized to be true local energy minima on the potential energy surfaces without imaginary frequencies. 

## 3. Results and Discussion

### 3.1. Molecular Structures

**Figure 2:**
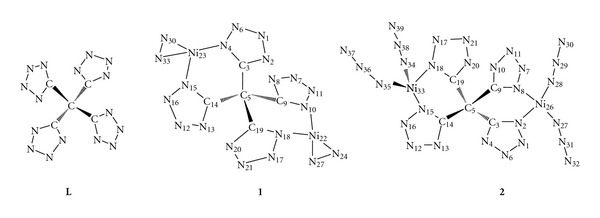
Optimized molecular structures of title complexes and ligand (hydrogen atom omitted for clarity).

Figure 2 shows the optimized structures of **1** and **2**, and the corresponding Ni–N bond lengths were listed in Table 1. The nickel(II) ion is tetrahedrally coordinated in both the complexes. The Ni(23) atom is coplanar with N(4), N(30), and N(33) in **1**, but N(15) is perpendicular to the plane. On the opposite side, the Ni(22) atom is coplanar with N(10), N(24), and N(27), with N(18) perpendicular to it. Similarly, Ni(33) atom is coplanar with N(34), N(35), and N(18) in **2**, with N(15) perpendicular to the plane. On the opposite side, Ni(26) atom is coplanar with N(8), N(27), and N(28), with N(2) perpendicular to it. 

As can be seen from Table 1, there is a difference in the Ni–**L **bond strength in **1** and** 2**. The coordinated bonds between Ni and N of N_2_H_4_ are over 2 Å in** 1**, while those between Ni and N of **L** are less that 2 Å. Ni atom in** 1 **combines with **L** more strongly than with N_2_H_4_, with **L** being deprotonated and deformed. On the contrary, the coordinated bonds between Ni and N_3_^−^ for **2 **are less than 2 Å, while those between Ni and **L** are larger than 2 Å. Ni atom in** 2 **combines with **L** less strongly than with N_3_^−^, and the structure of **L** hardly changes as compared to its uncoordinated one. As a whole, there are minor Jahn-Teller distortions in both **1** and **2**, with two long Ni–N lengths and two short ones. 

Observation of the geometrical structure of N_2_H_4_ and N_3_^−^ before and after coordination process found that the r(N–N) = 1.5571 and 1.5601 Å in hydrazine moiety, for **1 **are obviously larger than 1.4621 Å in the isolated N_2_H_4_ molecule. Also, the hydrogen atoms of hydrazine moiety for **1** prefer an eclipsed configuration in contrast to the stagger one of isolated N_2_H_4_ molecule (hydrazine). As for **2, **the four azides are nearly linear with the N–N–N angles being from 177.71° to 179.01°, indicating that the N–N–N angle hardly changes in the coordination process. The N–N bond lengths for the azide ligand in complex **2 **are 1.17 to 1.21 Å (the longer one neighboring with Ni), which are larger than 1.15 to 1.17 Å of the isolated azide acid. 

### 3.2. Infrared Spectroscopy

**Figure 3:**
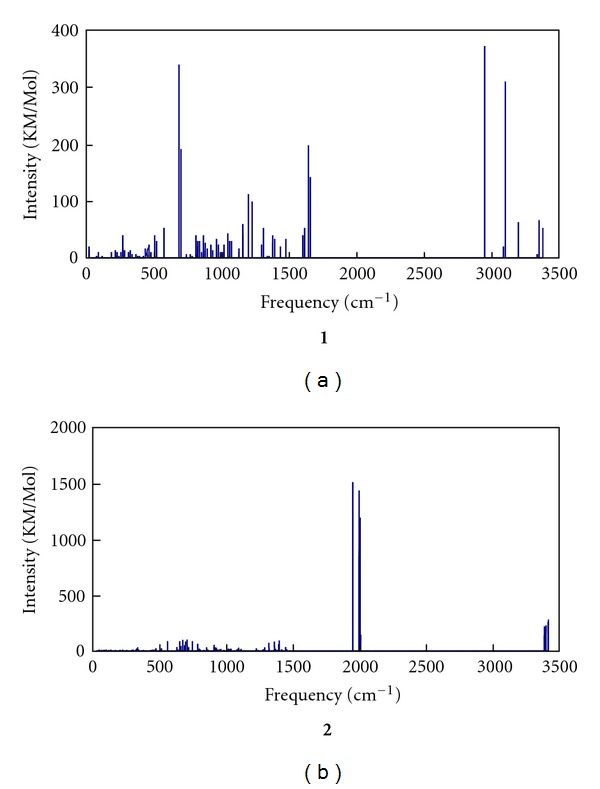
Simulated IR spectroscopies of title complexes.

Figure 3 showed the calculated IR spectroscopy for **1** and **2**. The vibrational frequencies produced by DFT-B3LYP were multiplied by a scale factor 0.89 [25]. For complex **1**, the 250 cm^−1^ mode is assigned to the cooperative rocking of the complex. Frequency around 630 cm^−1^ is associated with the H–N–H rocking modes for the N_2_H_4_ moiety and this mode exhibit very large intensities as a result of large dipole moment change. Frequency at 747 cm^−1^ is associated with the N–N stretching modes of a tetrazole ring. The 1067–1129 cm^−1^ modes are associated with the stretches of H–N–H wagging for the N_2_H_4_ moiety. The 1270 cm^−1^ mode is assigned to the C-C stretching of ligand **L**. The 2720–2900 cm^−1^ modes are of the N–H stretching of the N_2_H_4_ moiety with two strong peaks. For complex **2**, there are only two major peak regions at 1810–1860 cm^−1^ and 3140–3170 cm^−1^. The former modes associated with N–N stretching of the azide exhibit strong intensities. While the latter modes associated with N–H stretching on the tetrazole ring exhibits weak intensity. It should be noted that the intensities of strong peaks of **2** are much larger than those of **1**, since the N–N bond stretching of the azide moiety induces great change of the dipole moment for the former.

### 3.3. Stability Constants of the Complexes

The changes of the thermodynamic functions for reaction M + nL = ML_n_ were listed in Table 2. The stability constants of the complexes were then derived from the equation of Δ*G* = −*RT*lnK.

As can be seen from Table 2, the stability constant of **2** is larger than that of **1** at low temperature, while at high temperature, **2** is more unstable than **1**. Therefore, **2** is readily easy to synthesize compared to **1**. The stability constants for both the complexes decrease as temperature increases. There are inversion temperatures for the complexes at the range of 900–1200 K. The complexes become unstable above 1043 K and 985 K for **1** and **2**, respectively. Of course, the stability constants and the inversion temperatures refer to the metal-ligand bonds. 

### 3.4. Changes of Thermodynamic Properties in Combustion

The changes of thermodynamic properties in combustion were tabulated in Table 3. As can be seen from Table 3, the complex **2** releases more energy in combustion or explosion compared to **1**. With temperature increasing, the absolute values of Δ_c_ *H* increase. Of course, the actual energies being released would be less than the predicted values since the combustion enthalpies were evaluated on condition of rich oxygen. 

### 3.5. Energy Gap

The HOMO-LUOM energy gap could be regarded as the quantitative index in evaluating the impact sensitivity of energetic complexes with similar geometric structure. The less the energy gap is, the more sensitive the energetic complex is.  Table 4 listed the energies of HOMO and LUMO and their gaps. The energy gap of the complex **2** with azide ligand is as small as 2.37 eV. This is in good agreement with the fact that the metallic azide is widely used as initiator due to its high sensitivity. Judged by the large difference of energy gap between **1 **and **2**, it can be speculated that **1** is an insensitive explosive. 

## 4. Conclusion

DFT-B3LYP computations in combination with SDD basis set for Ni and 3-21+G* for nonmetallic atoms were performed on dinickel bishydrazine ter[(1*H*-Tetrazol-3-yl)methan-3yl]- 1*H*-tetrazole and dinickel tetraazide ter[(1*H*-Tetrazol-3-yl)methan-3yl]-1*H*-tetrazolate. Tetra(1*H*-Tetrazol-5-yl)methane was used to bridge two center metal ions Ni(II). The Ni(II) can coordinate tetrahedrally with tetra-tetrazolyl-methane and hydrazine/azide anion. The metal-ligand bonds are stable below 1000 K, judged from the stability constants. Both complexes release a great amount of heats in combustion. Complex **1**, coordinated with ligands of tetra(1*H*-Tetrazol-5-yl)methane and hydrazine, was predicted to have low sensitivity. 

## Figures and Tables

**Table 1:** Optimized bond length (Å) of Ni–N.

Compound 1		Compound 2	
Bond	Length	Bond	Length
Ni(22)–N(24)	2.0395	Ni(26)–N(27)	1.9155
Ni(22)–N(27)	2.0595	Ni(26)–N(28)	1.9295
Ni(23)–N(30)	2.0635	Ni(33)–N(34)	1.9345
Ni(23)–N(33)	2.0655	Ni(33)–N(35)	1.9585
Ni(22)–N(10)	1.9685	Ni(26)–N(2)	2.0175
Ni(22)–N(18)	1.9635	Ni(26)–N(8)	2.0625
Ni(23)–N(4)	1.9935	Ni(33)–N(15)	2.1085
Ni(23)–N(15)	1.9715	Ni(33)–N(18)	2.0265

**Table 2:** Thermodynamic properties and stability constants for **1** and **2** at various temperatures *.

Compound	TEMP/ K	ΔE/kJ/mol	ΔZPE/kJ/mol	Δ*H *_T_/kJ/mol	ΔH/kJ/mol	ΔS/kJ/mol	ΔG/kJ/mol	log K
	298.15	−689.92	−67.08	−17.07	−774.07	−0.71	−561.82	98.43
	600.00	−689.92	−67.08	−21.04	−778.04	−0.73	−338.22	29.45
1	900.00	−689.92	−67.08	−24.41	−781.41	−0.74	−111.57	6.48
	1200.00	−689.92	−67.08	−28.93	−785.93	−0.75	118.05	−5.14
	1500.00	−689.92	−67.08	−34.94	−791.94	−0.76	350.24	−12.20

	298.15	−1039.98	−15.96	−21.59	−1077.53	−1.07	−759.34	133.04
	600.00	−1039.98	−15.96	−25.07	−1081.01	−1.09	−428.30	37.29
2	900.00	−1039.98	−15.96	−24.21	−1080.15	−1.09	−96.01	5.57
	1200.00	−1039.98	−15.96	−22.51	−1078.46	−1.10	237.51	−10.34
	1500.00	−1039.98	−15.96	−21.31	−1077.26	−1.10	571.87	−19.91

*ΔH = ΔE + ΔZPE + ΔH_T_.

**Table 3:** Calculated changes of thermodynamic properties in combustion^a^.

Compound	TEMP/K	ΔE/kJ/mol	ΔZPE/kJ/mol	ΔH_T_/kJ/mol	Δ_c_ *H*/kJ/mol
	298.15	−3515.07	−154.08	62.72	−3606.43
	600.00	−3515.07	−154.08	56.71	−3612.44
**1**	900.00	−3515.07	−154.08	19.25	−3649.90
	1200.00	−3515.07	−154.08	−27.13	−3696.28
	1500.00	−3515.07	−154.08	−76.05	−3745.20

	298.15	−3541.21	−183.07	70.25	−3654.03
	600.00	−3541.21	−183.07	59.70	−3664.58
**2**	900.00	−3541.21	−183.07	14.72	−3709.56
	1200.00	−3541.21	−183.07	−39.64	−3763.92
	1500.00	−3541.21	−183.07	−96.62	−3820.90

^a^Combustion reactions: C_5_H_8_N_20_Ni_2 _ + 8O_2 _ = 5CO_2 _ + 4H_2_O + 10N_2 _ + 2NiO for complex **1**, and C_5_H_4_N_2_8Ni_2 _ + 7O_2 _ = 5CO_2 _ + 2H_2_O + 14N_2 _ + 2NiO for complex **2**.

**Table 4:** The frontier orbital energy and their gap.

Compound	HOMO/eV	LUMO/eV	Energy gap/eV
**1**	−2.48	−7.53	5.05
**2**	−3.71	−6.08	2.37
